# Comparative transcriptome and proteome analysis to reveal the biosynthesis of gold nanoparticles in Arabidopsis

**DOI:** 10.1038/srep21733

**Published:** 2016-02-23

**Authors:** Manish Tiwari, Sneha Krishnamurthy, Devesh Shukla, Jeffrey Kiiskila, Ajay Jain, Rupali Datta, Nilesh Sharma, Shivendra V. Sahi

**Affiliations:** 1 Department of Biology, Western Kentucky University, 1906 College heights, Bowling Green, 42101-1080 Kentucky, USA; 2 Department of Biological Sciences, Michigan Technological University, Houghton, Michigan, USA; 3 National Research Centre on Plant Biotechnology, Lal Bahadur Shastri Building, Pusa Campus, New Delhi, 110012, India

## Abstract

A large number of plants have been tested and exploited in search of a green chemistry approach for the fabrication of gold or other precious metal nanomaterials. Despite the potential of plant based methods, very little is known about the underlying biochemical reactions and genes involved in the biotransformation mechanism of AuCl_4_ into gold nanoparticles (AuNPs). In this research, we thus focused on studying the effect of Au on growth and nanoparticles formation by analyses of transcriptome, proteome and ionome shift in *Arabidopsis*. Au exposure favored the growth of *Arabidopsis* seedling and induced formation of nanoparticles in root and shoot, as indicated by optical and hyperspectral imaging. Root transcriptome analysis demonstrated the differential expression of the members of *WRKY*, *MYB* and *BHLH* gene families, which are involved in the Fe and other essential metals homeostasis. The proteome analysis revealed that Glutathione S-transferases were induced in the shoot and suggested its potential role in the biosynthesis AuNPs. This study also demonstrated the role of plant hormone auxin in determining the Au induced root system architecture. This is the first study using an integrated approach to understand the *in planta* biotransformation of KAuCl_4_ into AuNPs.

The use of metal nanoparticles in diverse areas has increased dramatically in recent years and, therefore, the development of safe methods for the fabrication of various shapes and sizes of nanomaterials has become a need and subject of extensive research. Application of metal nanoparticles includes mostly in photovoltaics, sensory probes, therapeutic agents, electronic conductors, diagnostics, drug delivery, and extend to food and agriculture sector as well^1^,^2^,^3^,^4^. The discovery of gold nanoparticles (AuNPs) application in the treatment of cancer and targeted drug delivery has opened the therapeutic potential of nanoparticles^5^. Having several unique physical and chemical properties, gold (Au) is one of the most studied metals for the synthesis of nanoparticles^1^. Due to wide use, the demand of nanoparticles including AuNPs is rising and projected to cross $1 trillion market by 2015^6^. Chemically, AuNPs are synthesized by the reduction of Au (III) (Chloroauric acid; HAuCl_4_) to Au (0) in water, and the reaction can be catalyzed by citrate. The methods of chemical synthesis of nanoparticles are well described but the major limitation of these methods is the generation of hazardous byproducts which can impact the environment negatively^7^. Therefore, nanomaterial fabrication based on plant or microbial systems is an eco-friendly method, and is considered as a means of sustainable development. Considerable progress has been made in the synthesis of AuNPs by using microorganisms and plant materials in the past decade. The various biological methods were developed for the synthesis of exquisite shapes and sizes of gold nanoparticles in past few years^8^,^9^,^10^.

Despite using different plant species for the harvest of AuNPs, the exact mechanisms underlying *in planta* reduction of Au or Ag are almost unknown. Due to lack of mechanistic details, these methods are less controllable and reproducible, and thus cannot be used for scaling up production at the commercial level. Few efforts have been made to identify the molecular entities involved in the acquisition of KAuCl_4_ by root, bioreduction of KAuCl_4_ to AuNPs *in planta*, or transport of those from root to shoot using model plants^11^,^12^,^13^,^14^. Studies based on examining the transcriptional perturbations associated with ionic Au exposure in *Arabidopsis* revealed the upregulation of a number of stress responsive genes such as glutathione transferases, cytochrome P450, peroxidases and ABA mediated signaling^11^,^12^,^13^. In addition, some investigations based on bacterial systems also revealed some insight into the mechanism of nanoparticle biofabrication^15^,^16^. However, knowledge from these studies is too far from being sufficient for designing an efficient system for bioprocess engineering.

The combined implication of *-omic* approaches such as transcriptome and proteome studies for elucidation of molecular processes including abiotic stresses has proved its worth in recent years. The integrative transcriptome and proteome analyses of nitrogen deficiency revealed the nitrogen sparing mechanism in *Chlamydomonas reinhardtii*^17^. An integrative analysis of the transcriptome and proteome of the pulp of a spontaneous late-ripening sweet orange mutant and wild type explored molecular insights of ripening related events in *Citrus sinensis*^18^. In the present study, we report the molecular overview of AuNPs formation in a model plant, *Arabidopsis thaliana*, using the integrative approach that includes comparative transcriptome, proteome and ionome profiling. We also report that auxin signaling might play a critical role in modulating the Au induced changes in root system architecture of *Arabidopsis*.

## Results and Discussion

### Morphological changes in *Arabidopsis* exposed to Au

**Figure 1:**
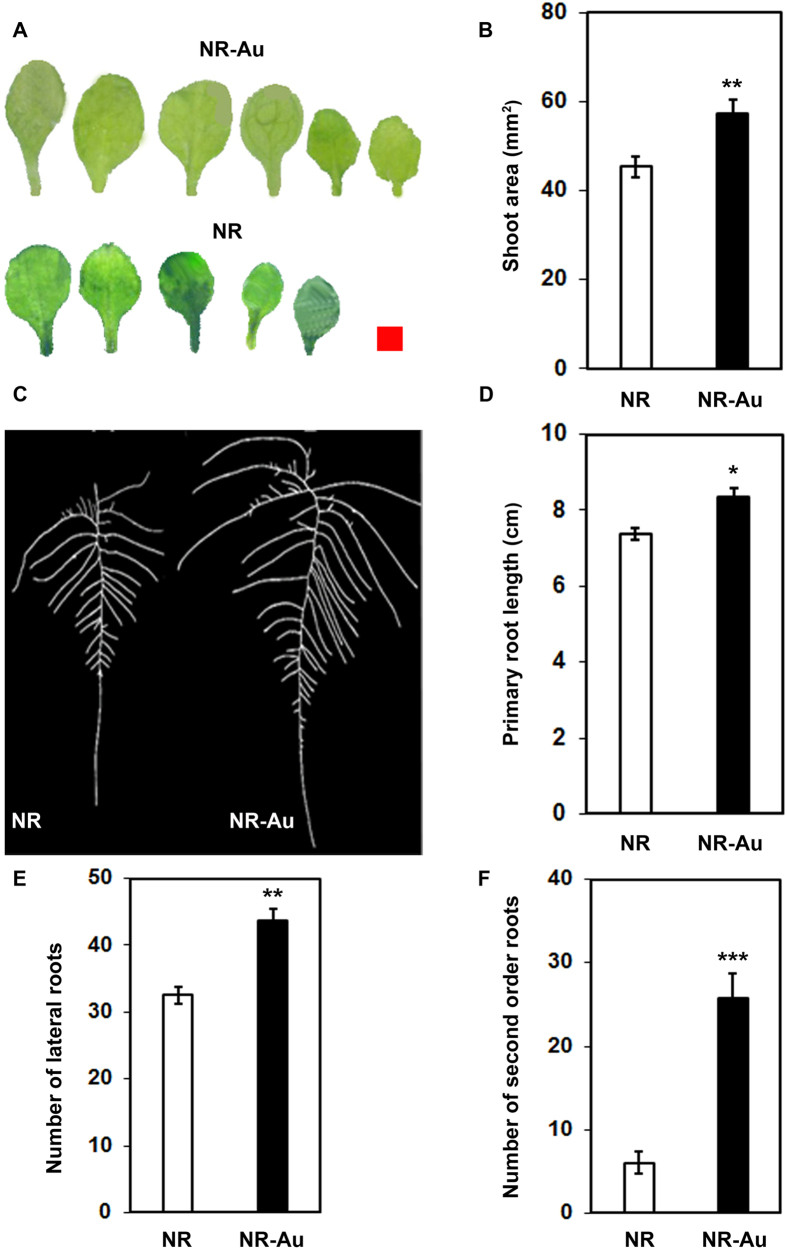
Effects of Au on morphology of Arabidopsis seedling. Five days old germinated plants transferred on nutrient rich MS medium (NR) and MS supplemented with 10 ppm Au (NR-Au) and allowed to grow for one week under hydroponic conditions. (**A**) Image showing all the rosette leaves of individual seedling grown under NR and NR-Au media. Red square is equivalent to 1 mm^2^ on scale. (**B**) Total shoot area of individual seedlings grown under indicated media. (**C**) Root system architecture of representative plant shown in (**A**). (**D**) Measurement of root parameters like primary root length, (**E**) lateral root numbers and (F) second order lateral root numbers. Data are presented as ± SEM (n = 12–15). Significance was determined by Student’s unpaired t-test P ≤ 0.05–0.001. *, ** and ***represent significantly difference from NR (P > 0.01, P > 0.001 and P > 0.0001 respectively).

To examine developmental changes in presence of Au, five-days-old seedlings of *Arabidopsis* were subjected to Au treatment (KAuCl_4_) for one week under hydroponic growth condition. The examination of root system architecture (RSA) revealed that primary root length was significantly increased upon exposure of Au compared to the control (Fig. 1C,D). Similar to the primary root, the number of first and second order lateral roots was substantially higher in control plants when Au (10 ppm) was present in the media (Fig. 1E,F). A comparable and positive effect of Au on aerial parts of plant was also observed (Fig. 1A,B). Leaves of Au-dosed seedlings were larger in size to that of control plants, and total shoot area of individual seedling was noticeably increased. The positive changes in RSA and shoot in this experiment are in agreement to the previous report that showed the stimulatory effect of Au on primary root length up to 10 ppm concentration in *Arabidopsis*^14^. In general, RSA reflects adaptability of plants in response to the nutrient status. Plant senses environmental cues in form of nutrient excess or deprivation and integrates signal to maintain root system architecture to face the challenges^19^. A plethora of reports revealed that various nutrients are differently assessed by plants, and subsequently particular genes and uptake system interact with corresponding nutrient or element^19^,^20^. The compute system of plant for measuring nutrient imbalance upon local or systemic nutrient deficiency response is highly specific and activates particular acquisition system, which may alter the RSA to acquire more nutrient^19^. Therefore, positive change in RSA of *Arabidopsis* could be indirect consequence of Au exposure which might be resulted by deficiency response of some essential nutrients.

### Detection of AuNPs in Arabidopsis

**Figure 2:**
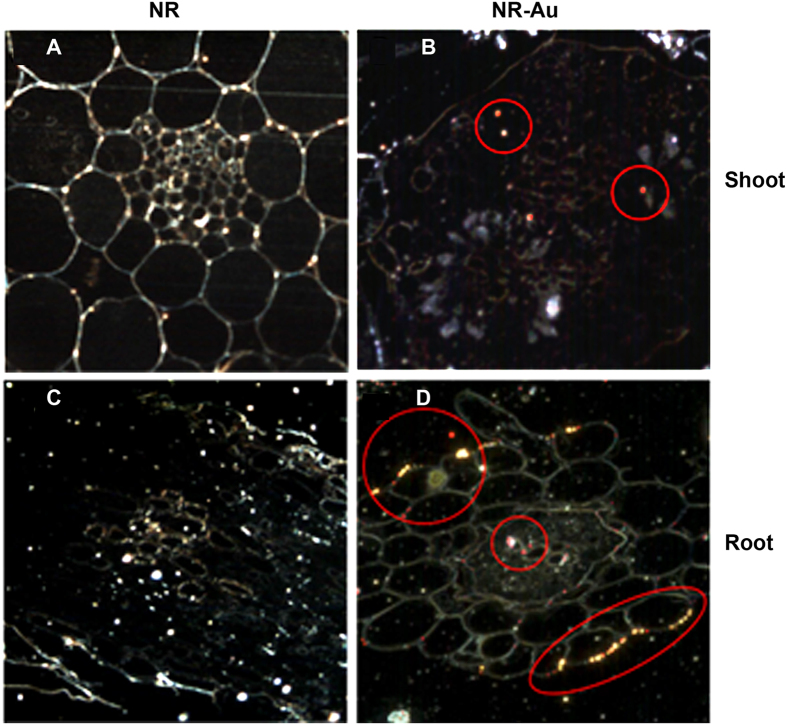
High contrast dark-field imaging for detection of AuNPs in tissues of Arabidopsis. Images were taken in root of untreated seedling (**A**), Au (10 ppm) exposed root (**B**), control shoot (**C**) and Au (10 ppm) exposed shoot (**D**). Red spots display the distribution of AuNPs in cells and marked with red circle for better visualization.

In order to determine formation of AuNPs in plant tissues, subcellular detection of the AuNPs was carried out through high contrast dark-field imaging by CytoViva microscopy. The application of hyperspectral spectroscopy has proven its usefulness in localization of nanoscale materials in biological systems^21^. This kind of imaging can be accomplished without the staining of samples. The hyperspectral imaging was performed in both root and shoot tissues of treated and untreated plants (NR) grown under same conditions. The captured signal intensities in the treated samples analyzed against control confirmed the presence of AuNPs (Fig. 2B,D). The bright red spots in Au-dosed samples indicated presence of the AuNPs in *Arabidopsis* root and shoot (Fig. 2). The results were further verified through detailed spectral analysis. Moreover, to investigate the effect of varying Au concentrations on AuNPs synthesis, the experiments were performed using 10 ppm, 25 ppm and 50 ppm of KAuCl_4_, which demonstrated a dose-dependent synthesis of AuNPs in both root and shoot (data not shown here). A correlation between formation of AuNPs and AuCl_4_^−^ concentration occurred up to the 50 ppm, however, increasing concentrations of AuCl_4_^−^ led to the negative growth in *Arabidopsis*. Therefore, 10 ppm KAuCl_4_ was selected for transcriptomic and proteomic studies.

### Root transcriptome analysis and annotation under Au exposure

To investigate the role of genes involved in the biotransformation of AuCl_4_^−^ into AuNPs, a transcriptome analysis of the root was performed using microarray. The analysis identified differential expression of 325 genes (more than 2-fold) responsive to the Au. Gene Ontology (GO) annotation of differentially expressed genes (DEGs) was carried out. The enriched GO obtained by functional categorization for cellular component revealed the maximum number of genes belonging to nucleus (88), cytoplasmic component (71), extracellular (67) and other membranes (56) (Supplementary Fig. S1A). The functional classification of GO term for biological process indicated that largest number of genes were related to cellular processes (190), other metabolic processes (172), responses to stress (121) and abiotic or biotic stimulus (100) (Supplementary Fig. S1B). The contribution of other binding proteins (111), proteins with other enzyme activities (59), transferase activities (40), transporter activities (25) and unknown molecular functions (59) was high in functional categorization of all GO terms.

**Figure 3:**
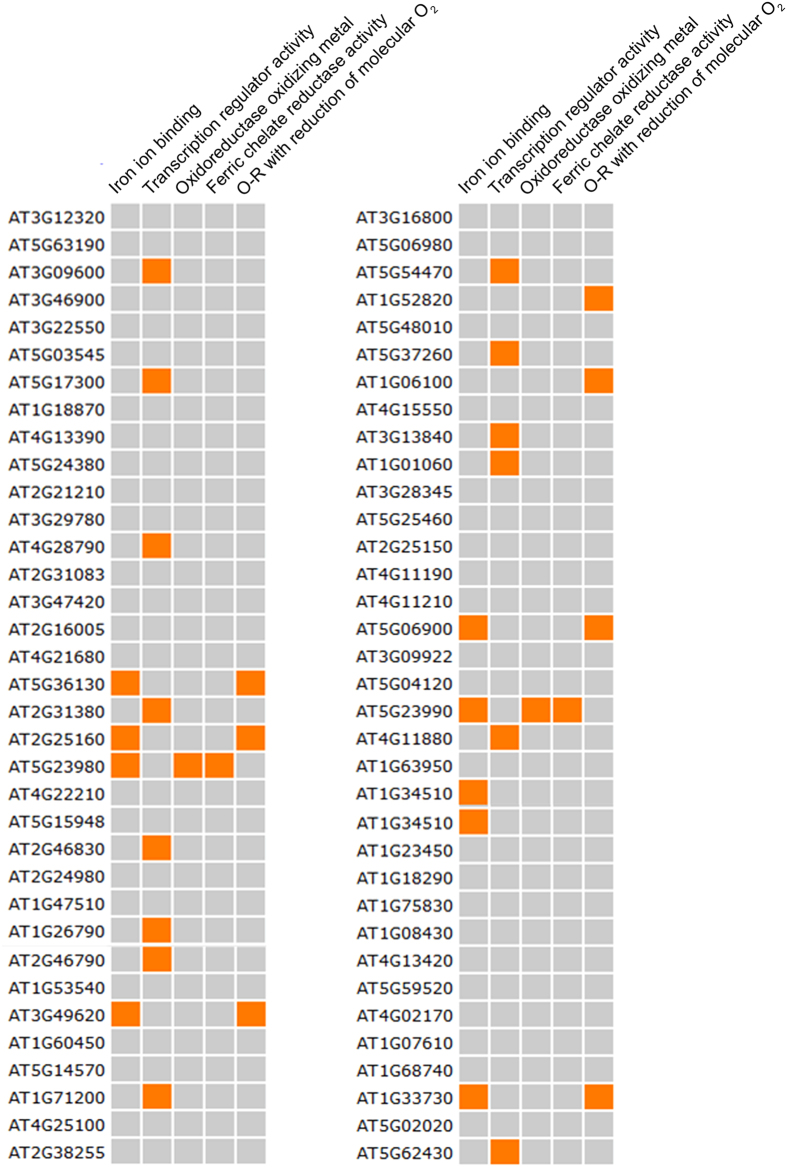
Plant Gene Set Enrichment Analysis of significantly upregulated genes.

GO slim molecular function annotation of all upregulated genes through Plant Gene Set Enrichment Analysis (Plant GSEA) revealed 9 genes related to Fe ion binding and 2 for Fe chelate reductase activity (Fig. 3 and Supplementary Table 1). The analysis suggested that number of genes related with Fe homeostasis had altered by Au treatment. Consistently, a large number of genes belonging to oxido-reductase family (9) were upregulated. Based on the type of reaction catalyzed, these genes can be further divided into two classes: first group had oxido-reducatse activity by acting on paired donors with incorporation or reduction of molecular oxygen, while others were involved in the oxidation of metal ions with NAD or NADP acting as an acceptor molecule (Supplementary Table 1). Gene singular enrichment analysis indicated the abundance of ‘transcription regulator activity’ term among upregulated genes (14) (Supplementary Table 1). This group consisted transcription factors like *MYB*, *bHLH*, *MADS* domain protein, *Clavata* and *Dof* zinc finger protein, which regulate diverse developmental programs in *Arabidopsis*. Unlike upregulated genes, GSEA analysis of downregulated genes revealed the largest number of loci (64) grouped under GO term ‘binding’ consisting of Fe, transition ion, cation and metal binding (Supplementary Table 2). The catalytic activity was other major GO term, which contains genes for enzymatic activity such as peroxidase, oxidoreductase, chitinase and different kinases.

### Majority of metal responsive and binding genes were upregulated

Amongst differentially expressed genes, 70 genes were upregulated up to two fold in root (Supplementary Table 3). The classification of upregulated genes based on metal responses indicates that 12.46% genes were associated with cation binding (Supplementary Fig. S2). The expression of *ferric reduction oxidase 5* (*FRO5*) was highest (17.53 fold) among upregulated genes. The induced loci encode different types of transporters such as copper transporter, nitrate transporter, ABC transporter, heavy-metal-associated protein (*HMA*), zinc (Zn) transporter, malate transporter and phosphate transporter. These genes are responsible for uptake of essential elements and nutrients such as Fe, Cu, Zn, NO_3_ and PO_4_^22^,^23^,^24^.

Root transcriptome analysis indicated that Au exposure created metal deficiency responses as evidenced by upregulation of genes responsive to Fe, Zn, Cu and Mn. The Fe homeostasis was much affected by Au as evident by the upregulation of multiple Fe uptake regulators, for instance, *FRO4*, *FRO5*, *COPT2*, and *YSL2* obtained in microarray. Being a cofactor of heme, Fe is the most required element for redox maintenance of cell. Heme is a constituent of cytochrome P450s, Fe (II)-dependent oxygenase and some peroxidases engaged in oxido-reductase activities. It is likely that Au builds up oxidative stress and, to cope with that, plant activates its signaling cascade eventually boosting Fe acquisition by upregulating genes of Fe uptake and Fe binding proteins. The functions of few HMA domain containing proteins are well studied during vascular loading of Zn and Mn in the root^23^. The upregulation of *superoxide dismutase* (*SOD*) suggested that SOD actively scavenges the free radicals induced by Au in *Arabidopsis*.

Many DNA binding transcription factors were significantly induced after Au treatment. The microarray data revealed the upregulation of 2 bHLH transcription factors (Supplementary Table 3). The basic helix-loop-helix (bHLH) transcription factor is a heterodimeric protein and its activity is highly regulated by dimerization of the subunits. The bHLH acts as transcriptional activators in conjunction with other factors in cooperative manner, and controls several processes including Fe uptake^25^. Two members of *Dof*-type zinc finger were also found to be upregulated in expression studies. Dof domain proteins have dual functions either as transcriptional activator or repressor, and thus play a critical role in maintaining photoperiodism, plant growth and development^26^. The MYB domain containing proteins were other important upregulated transcription factors (Supplementary Table 3). *Reveille1* (*RVE1*) is a member of MYB family which participates in auxin biosynthesis in *Arabidopsis*^27^. A significant high expression of genes associated with the circadian clock, namely *CCA1*, *LHY*, *RVE1*, *RVE2* and *LNK3* was also observed in microarray data (Table 1 & Supplementary Table 3). The *CCA1* is central component of circadian clock which regulates rhythmicity by integrating several factors including *LHY*, *RVE1*, *RVE2* and *Light-inducible and clock-regulated 3*^28^. These genes function synergistically in a way to modulate the circadian clock and flowering; and it is presumed that Au responsive imbalance in nutrient homeostasis would have triggered their induced expressions in *Arabidopsis*. The temporal coordination of ROS signaling by *CCA1* and the reciprocal control of circadian output by ROS reveal a mechanistic link that allows plants to master oxidative stress responses^29^. Moreover, ROS is a major byproduct of Au stress as manifested by induction of a number of ROS scavenging genes. The available reports indicate that environmental stresses modulate abundance of circadian transcripts or its interacting factors in *Arabidopsis*^29^,^30^,^31^. Altogether, microarray data revealed that a large number of metal related genes and transcription factors controlling growth-development were induced by Au.

### Stress and defense related genes were downregulated by Au exposure

Microarray results showed that 118 genes were downregulated up to 2.5-fold change (Supplementary Table 4). Cytochrome P450 and germin-like protein (GLPs) were the most downregulated gene families representing with 5 and 7 members, respectively. Cytochrome P450s are the class of heme binding oxidase involved in a wide range of metabolic reactions of cell leading to synthesis of fatty acid, hormone, secondary metabolites and defense compounds^32^,^33^,^34^. Numerous reports exposed the important role of GLPs in maintaining the basal immunity by affecting the defense process in plants^35^,^36^. Many loci encoding stress marker enzymes, namely, peroxidase, serine carboxypeptidase-like 14, chitinase class 4-like protein, aspartyl protease family protein, glycosyl hydrolase, phospholipase-A 2A, alternative oxidase3 and subtilase were downregulated^37^,^38^,^39^. The additional defense related genes such as TIR-domain-containing protein, *TIR-NBS-LRR* class protein, *LRR-protein kinase* and *serine threonine-protein kinase* were also notably downregulated (Supplementary Table 4). These kinases mediate downstream signaling of the defense response by interacting itself or with the effector of pathogens^40^. The expression of loci such as *RING-H2* zinc finger protein, *WRKY* transcription factor (*WRKY 59* and *WRKY 62*), *NAC* domain-containing protein 42 (*ANAC042*) and ethylene-responsive transcription factor 13 (*ERF 13*) was also found significantly reduced after Au treatment. The *WRKY* transcription factors are well studied for their role in salicylic acid-induced disease responses.

Intracellular calcium (Ca) maintaining genes, for example Ca^2+^-transporting ATPase and calmodulin binding protein, and three genes encoding Cu transport proteins and cupredoxin superfamily protein were downregulated. Calcium acts as secondary messengers in several signal transduction processes, and cell invests huge energy to maintain its steep gradient between intra- and extracellular space^41^. Interestingly, five glutathione S-transferases (GSTs), representatives of lambda and phi classes of GST family, were substantially downregulated in root (Supplementary Table 4). The observation contradicted our previous report which showed that many GST members were upregulated in root during short-term Au exposure^13^. It is possible that a longer Au exposure (seven day) affects GSTs differently as seen in this study. A *cis*-regulatory motif analysis of promoters of all GSTs was performed in order to get more molecular insight since we suspected the potential role of GSTs in AuNPs synthesis. The presence of W-box in promoter of every GSTs signals that WRKY transcription factors play a crucial role in the downregulation of a large number of defense related genes (Supplementary Table 5). The DNA-binding domain of WRKY transcription factors recognizes the W-box motif in promoter region. This assumption is further strengthened by the presence of *WRKY69* and *WRKY52* in significantly downregulated list of genes (Supplementary Table 4), which are well known for having their role in disease resistance in *Arabidopsis*^42^. Similar to *WRKY*, a repressed expression of *ANAC042* and *RING finger E3 ligase*, which participate in defense and salt stress respectively, might be the reason for the downregulation of a large number of defense related genes^43^,^44^. Overall, these observations explicitly indicated that Au had a negative impact on the expressions of stress and basal immunity related genes in *Arabidopsis*.

### Quantitative RT-PCR validation of microarray data

**Figure 4:**
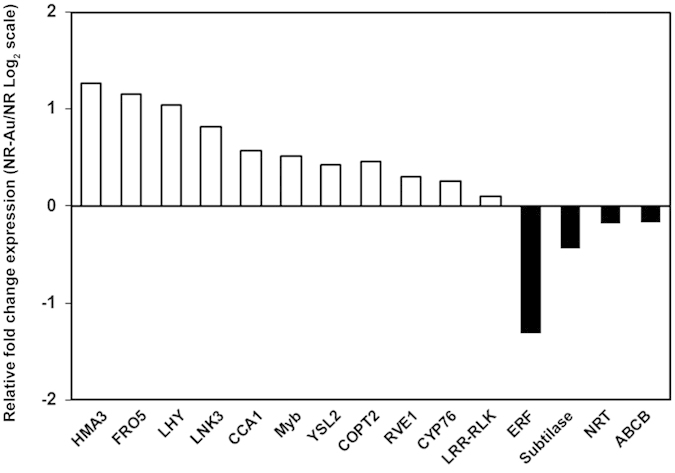
Expression of genes in the shoot of *Arabidopsis* after Au treatment.

In order to validate microarray results, qRT-PCR analysis of selected genes was carried out. The criteria for gene selection was mainly the fold change expressions and molecular significance (Table 1). *Subtilase*, *LRR-RLK* and *ERF13* were selected from downregulated list for expression analysis. Quantitative RT-PCR observations revealed that genes expressions followed the same trend as observed in microarray experiment with little variation in fold change (Table 1). Further, we have also measured the expression of genes under the treatment of 25 ppm Au in order to know the effect of increasing Au concentration. The results showed that expression of *CCA1*, *HMA3*, *ABC transporter B*, *LNK3* and *YSL2* was repressed at 25 ppm Au in contrast to induced expressions seen at 10 ppm Au (Table 1). The gene expression is a highly coordinated and spatiotemporally regulated process that can be changed differentially with elevated Au. We also measured the expressions of these genes in shoot tissue, and interestingly, found a similar expression pattern as seen in root except in case of *NRT* (Fig. 4). Thus, observation shows that qRT-PCR expression data largely followed the microarray gene expression pattern.

### Auxin responsive and related genes were induced

The positive change in morphology of *Arabidopsis* seedling occurred with Au treatment. The expression studies demonstrated that *CCA1*, *LHY*, *RVE1* and *RVE2* were induced significantly (Table 1 & Supplementary Table 3). These genes constitute components of circadian clock and are also positively regulated by plant hormone auxin. Moreover, some additional auxin responsive genes such as *small auxin upregulated RNA6* (*SAUR6*), *Inositol polyphosphate 5-phosphatase 11* and *ATP-Binding Cassette B15* were found to be upregulated by Au exposure (Supplementary Table 3). The expression of *SAURs* are transiently induced by auxin and some members for instance, *SAUR39* reported to affect the free auxin level and transport in *Arabidopsis*^45^. *Reveille1*, a homolog of *CCA1*, controls auxin level by promoting free auxin production in *Arabidopsis* instead of controlling rhythmicity^27^. It is important to mention that all these described genes are auxin responsive. In addition, we also found some genes related with auxin biosynthesis and signaling in gene expression analysis. Based on these observations, auxin seems to be responsible for Au induced changes in RSA of *Arabidopsis*. Plant hormones such as auxin, cytokinin and ethylene have been known to regulate root development although the impact of auxin on the lateral roots development is more pronounced^46^,^47^. Thus, it is evident that auxin signaling cascade might play a major role in determining the Au induced changes in root system of *Arabidopsis*.

### Proteome modulation in response to Au treatment

**Figure 5:**
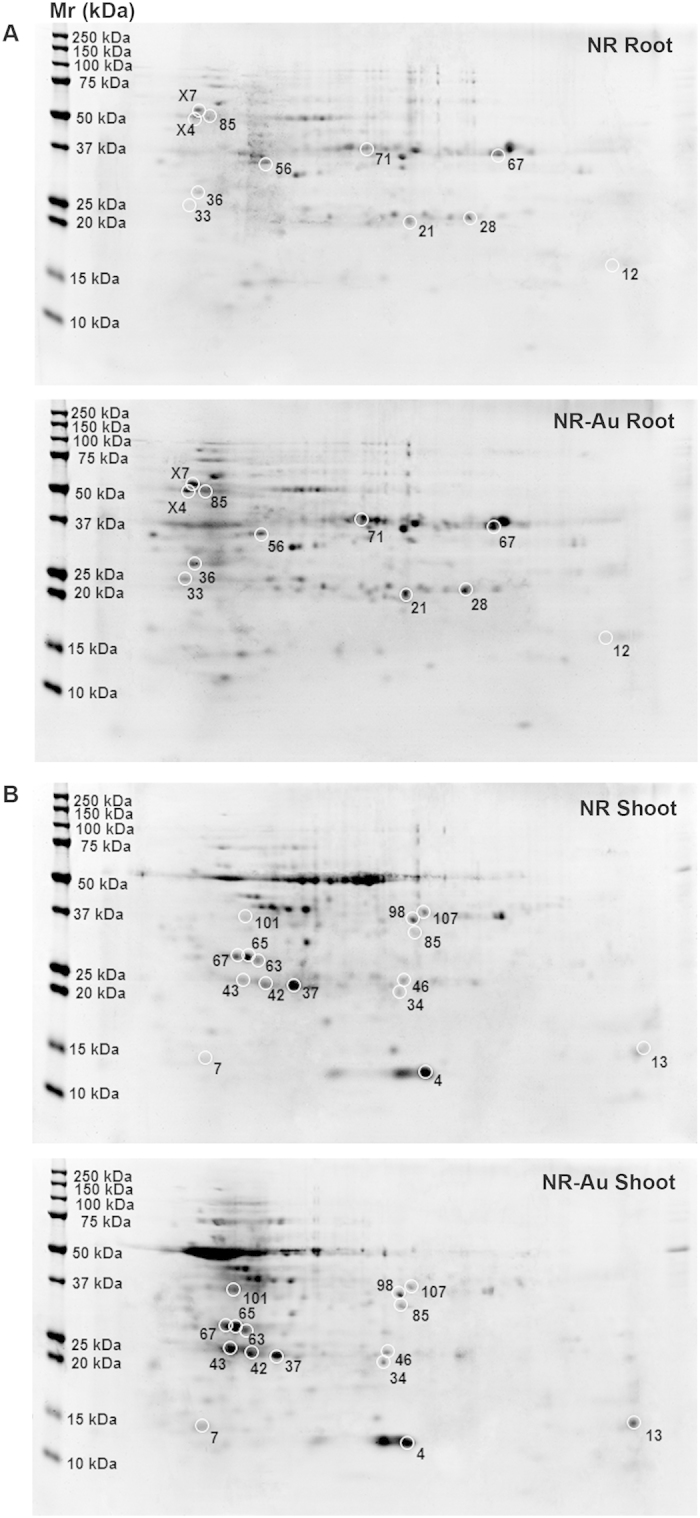
The 2D gel analysis of proteins extracted from root and shoot. The numbers assigned to the proteins spots correspond to listed in Table 2. The 2D gel electrophoresis picture showing the root (**A**) and shoot (**B**). The protein spots with significant differential expressions used in MALDI-TOF MS analysis were encircled.

Toward understanding the metal related regulatory processes in plants, several transcriptomics based efforts were made in recent years. However, it is equally accepted that gene expressions at a transcription could not always transduce up to the protein synthesis^48^. Hence, a quantitative proteomic analysis adds improvement to know the molecular events and assists in the identification of key regulators^49^. The comparative proteome analysis is now a common way to reveal the likely role of specific proteins in response to various abiotic and biotic stimuli in plants. To know the effect of Au on proteins and to establish correlation between transcriptome and proteome, a comparative proteome analysis of Au-treated root and shoot was performed. The two dimensional gel analysis identified 110 spots from root and 55 spots from shoot that showed up to 2-fold expression change (Fig. 5). Of these, the promising 10 spots from root and 15 spots from shoot were chosen for further identification based on relative intensity (Fig. 5). Some proteins from cellular metabolism such as glyceraldehyde-3-phosphate dehydrogenase-C subunit (GAPC-1), NADH dehydrogenase 1, 40S ribosomal protein S, glycerophosphodiester phosphodiesterase (GDPD3) and fructokinase-1 were induced (Table 2). In general, GAPC-1 is known for role in the glycolytic pathway, but at the same time, it can interacts with H_2_O_2_ thus becoming the part of signaling cascade of ROS^50^. The alteration of several proteins involved in primary metabolism suggests that Au exposure had moderately impacted carbon metabolism. These include the upregulation of GAPC1, 3-hydroxyisobutyrate dehydrogenase-like 3 (from pentose phosphate shunt) and fructokinase-1 in root (Table 2). Mn superoxide dismutase-1 (MSD1), a ROS scavenging protein, was downregulated in root. The MSD1 activity is critical for maintaining ROS localization which is important for embryo sac development in *Arabidopsis*^51^. This observation further supported that Au exerted oxidative stress by reacting with cellular proteins and enzymes and subsequently generated free radicles and ROS. The disruption or malfunction of electron transport system in mitochondria and chloroplast by Au, when it moved inside the cells, could be a probable mechanism of ROS production. The proteome study revealed the upregulation of mitochondrial proteins like NADH dehydrogenase (ubiquinone)-1 beta subunit-9 and cytochrome c oxidase subunit 5b-1 in shoot, which constitute the major component of electron transport chain. A number of oxidative stress regulated proteins such as GSTs, two thioredoxin like proteins and lactoylglutathione lyase (glyoxalase-I), were upregulated. As anticipated, redox maintaining cellular proteins, for instance, 3-hydroxyisobutyrate dehydrogenase and cytochrome c oxidase, were induced after Au exposure (Table 2). An interesting outcome of proteome study was the induction of HMA3 (7.98 fold). AtHMA3 is located on tonoplast and plays a role in the detoxification of Zn and Cd by vacuolar sequestration^23^. Among all 15 identified proteins in the shoot, only one protein, serine racemase, was downregulated (−3.44 fold). Serine racemase catalyzes D-serine to L-serine enantiomer and require Mg as co-factor for its activity^52^. The other upregulated protein identified was indole-3-acetic acid inducible 5, which is implicated in initiation of lateral roots in *Arabidopsis*^53^. The four GSTs have been identified in the proteome analysis. In agreement to the transcriptome data, one member of GST family, GST F6, was decreased in root whereas a significant rise in GST F2, GST U15, and GST U21 was noticed in the shoot. The detection of a large numbers of GSTs in shoot proteome is particularly important in light of higher Au accumulation in shoot. The function of GSTs in redox maintenance, xenobiotic detoxification and flavonoids subcellular transport is well-known. It is likely that induction of multiple GSTs is associated with the biotransformation of Au ions into AuNPs to protect cells from associated toxicity.

### Elemental profile of essential nutrients in seedlings exposed to Au

**Figure 6:**
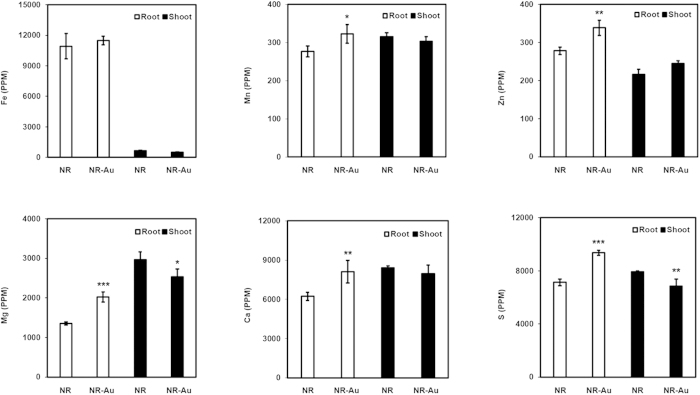
Quantification of essential metals in root and shoot of *Arabidopsis*. NR denotes for nutrient rich MS media whereas NR-Au for media supplemented with 10 ppm Au. Significance was determined by Student’s unpaired t-test P ≤ 0.05–0.001. ***and ***represent significantly difference from NR (P > 0.01, P > 0.001 and P > 0.0001 respectively).

While Au-induced transcriptome and proteome analyses indicated the alteration in the expression of metal responsive genes, it was worthwhile to measure content of Mg, Ca, Fe, Mn, Zn and S in root and shoot. The elemental profiling revealed that Fe was slightly higher in root under Au treatment as compared to control (Fig. 6). In contrast, a significant decrease in Fe was seen in shoot after Au treatment. Interestingly, it should be noted that this trend was seen for Mg, Ca, Fe, Mn, and S except Zn (Fig. 6). The observations from elemental analysis demonstrated that Au exposure caused a slight elevation of these elements in root, but at the same time, a significant decline occurred in shoot. From the foregoing account, it can be concluded that Au had negative impact on root to shoot translocation of these nutrients despite leading their accumulations in root.

## Conclusion

The precise biochemical and molecular steps underlying the biosynthesis of nanoparticles in plants are almost unknown. This study is an effort to understand these mechanisms by using a combination of -*omic* approaches. We investigated the effect of Au on growth and mechanism of AuNPs synthesis in *Arabidopsis* by using integrated approach of transcriptomics, proteomics and ionome profiling. We identified GSTs as a potential gene that might contribute to AuNPs formation and could be a possible target to manipulate AuNPs biosynthesis in future. In addition, we also report that Au interacts with essential elements such as Fe, Mn and Zn, and affected their normal homeostasis. We further proposed that plant hormone auxin plays a critical role behind Au-induced positive root growth and RSA change in *Arabidopsis*. Reconciled to all, this study shed some light and widened our understanding of molecular mechanisms related with AuNPs formation in *Arabidopsis*, which will be useful in the future for tailoring AuNPs of precise geometries.

## Methods

### Plant materials, growth conditions and Au treatment

*Arabidopsis thaliana* (Col-0) was used in this study. Plants were grown in tissue culture room under standard conditions that is 16-h light/8-h dark photoperiod, 22 °C and 140 to 160 μmol m^−2^ s^−1^ light intensity maintained by white florescent tubes. *Arabidopsis* seeds were surface-sterilized by 70% (v/v) ethanol for 3 min and 2% NaClO containing 0.05% Tween 20 for 5 min, and then washed 5 times with sterile water. For stratification, seeds were kept at 4 °C for 48 h in dark. Surface sterilized seeds were spread on polyproplylene mesh and allowed to grow for 5 day nourished with half-strength liquid MS medium under sterile hydroponic conditions devised in Magenta box^14^. After 5 days growth, the nutrient media was changed with fresh MS media containing 10 ppm KAuCl_4_ (NR-Au) and without Au (NR).

### Root growth measurement

The root and shoot of control and Au exposed seedlings were separated with each other. The parameters of root growth such as length of primary root, number of first and second order lateral roots of each seedling were measured using Image-J program. Similarly, the area of all leaves of rosette, represented as shoot area, was recorded. The photograph of individual seedlings were taken under the light Microscope (Leica MZ16, Switzerland).

### Sample preparation and detection of AuNPs

Small sections of root and hypocotyl were prepared and tissues were transferred in vial containing 3% glutaraldehyde in sodium cacodylate buffer (175 mM and pH 7.4), incubated for 1 hour and rinsed thrice with fresh phosphate buffer (0.06 M). The tissue was suspended with 1% OsO_4_ in phosphate buffer (0.12 M) and incubated in dark for 4 hours. The samples were rinsed with phosphate buffer (0.06 M) and distilled water three times, and dehydrated using 50%, 65%, 75%, 85%, 95% and 100% ethanol for 15 min to each. The dehydrated samples were infiltrated in Spurr’s epoxy resin gradually in 33%, 66%, 95%, and thrice in 100% resin (1hour each) and then left overnight in 100% resin. The sample pipetted into fresh resin in BEEM capsules (BEEM Inc., West Chester, PA, USA) and hardened at 70 °C for 18 hour. Ultrathin sections (approximately 200 nm thicknesses) were prepared using an ultramicrotome (Leica EM UC6) and mounted on clean microscopic slide.

For AuNPs detection, CytoViva’s hyperspectral imaging system was used, and optical as well as hyperspectral images were captured at 60× magnification. The hyperspectral data representing the light scatter from the samples were collected in a “push broom” approach. The spectral data was then presented as a data cube containing spectral and spatial data. The data cubes were spatially cropped to eliminate the background. Since the gold was synthesized in planta, the spectral library representing the AuNPs was built by gathering spectra within a particular intensity range in the 50 ppm root image using the Particle Filter (PF) tool. To eliminate any spectra that were not characteristic of AuNPs, the spectral library was filtered against the negative control hyperspectral image. Using the Spectral Angle Mapper (SAM) tool, the spectral library representing AuNPs was compared against every pixel in hyperspectral image. The SAM algorithm provided spatial and quantitative data by spectrally identifying any pixels whose spectral curves matched a spectrum in the library with a 10% margin of error.

### RNA isolation, Microarray hybridization and data analysis

Total RNA was isolated using Qiagen^TM^ RNeasy Plant Mini Kit. RNA was subjected to DNase treatment (Promega) for removing any trace amount of DNA. Microarray experiment was performed using Affymetrix GeneChip® ST 1.0 array GeneChips (Affymetrix, CA, USA). Amplified RNA preparation, hybridization to arrays, washing, staining, and scanning were carried out following the manufacturer’s instructions (Affymetrix, USA). Three independent biological replicates of control (NR) and Au dosed (NR-Au; 10 ppm KAuCl_4_) were taken. Image files were analyzed and the probe intensity (.cel) files were generated using the default setting of AGCC version 4. The intensity (.cel) files were normalized using RMA algorithm (Partek Genomics Suite 6.6, St. Louis, MO, USA). One-way ANOVA was applied and a step-up FDR p-value was calculated to sort out the statistically significant up/down regulated genes by filtering the data at p < 0.1 (FDR) and >2-fold change in the expression.

### Gene Ontology annotation and bioinformatic analysis

The GO term enrichment and functional categorization for biological process, cellular and molecular function was carried out using Gene Ontology at TAIR (www.arabidopsis.org/). The plant gene set enrichment analysis of differentially expressed genes (DEGs) was performed through online webserver Plant GeneSet Enrichment Analysis Tool kit under default parameter as Fisher Test statistical test method and cutoff value 0.05^54^. For regulatory *cis*-motif analysis, 1 kb long upstream sequences of each GSTs have been retrieved from The Arabidopsis Information Resource (TAIR) database (www.arabidopsis.org/), and searched for motif scan using online web tool Plant Cis-acting Regulatory DNA Elements (PLACE; www.dna.affrc.go.jp/PLACE/).

### Quantitative RT-PCR

The total RNA was treated with RQ1 RNase-free DNase (Promega) and 3 μg RNA was reverse transcribed using the SuperScript III first-strand synthesis kit (Invitrogen). The quantitative RT-PCR reaction was set up in 10 μl total volume which contains 5 μl of 2X SYBR (ABI Biosystems, USA), 1 μl of the five times diluted cDNA, 1 μl of forward and reverse primer (10 pM) and 2 μl nuclease free water. The thermal cycling parameters of reactions were performed under the conditions of: 10 min at 95 °C for initial polymerase activation, 15 sec at 95 °C for denaturation, 1 minute at 60 °C for anneal/extension and 40 cycles in a 96-well reaction plate in Applied Biosystems 7300 thermal cycler (Applied Biosystems, USA). To normalize the expression data, beta tubulin was used as an endogenous control and the relative expression levels of the gene was calculated by 2^–ΔΔCt^ method^55^. Most of the primers were designed from intron spanning region, wherever possible, using NCBI primer design tool. The combination of primer sequences used in this study are listed in Supplementary Table 6.

### Protein extraction

For differential proteome analysis, three independent biological replicates of root and shoot tissues of control and Au treated plants were performed in this study. Protein was extracted following trichloroacetic acid/acetone method^56^. Briefly, shoot tissue (150 mg) was homogenized in liquid nitrogen and incubated in 1.0 mL precipitation solution [10% TCA in acetone with 0.07% 2-mercaptoethanol (2-ME)] for 45 minutes at −20 °C. After centrifugation, pellet was washed several times in 1.0 mL of chilled acetone wash solution (acetone with 0.07% 2-ME). Pellet was dissolved in a urea-based extraction buffer [8.0 M Urea, 2.0 M Thiourea, 2% 3-cholamidopropyl dimethyl ammonio propanesulfonate (CHAPS), 1% Dithiothreitol (DTT) and 1.0 mM Phenyl methane sulfonyl fluoride (PMSF)], and then sonicated for 30 minutes. For root protein isolation, 150 mg of homogenized tissue was dissolved in a Tris-based extraction buffer [50 mM Tris-HCl, 2.0 mM Ethylene diamine tetraacetic acid (EDTA), 150 mM NaCl, 10% Glycerol, 0.1% Triton X-100, 1.0 mM PMFS and 1X Protease Inhibitor Cocktail (Thermo Scientific, Halt™)]. Protein was isolated following sonication and centrifugation. Protein concentrations were estimated using the Bio-Rad Protein Assay (Bio-Rad Laboratories), and quality was assessed by sodium dodecyl sulfate-polyacrylamide gel electrophoresis (SDS-PAGE).

### Two-dimensional electrophoresis and peptide mass fingerprinting

Each protein samples (300 μg) were prepared by using a ReadyPrep 2-D Cleanup Kit (Bio-Rad Laboratories, USA). The isoelectric focusing was done using PROTEAN™ IEF Cell (Bio-Rad Laboratories, USA) and immobilized pH gradient (IPG) strips with linear pH range (11 cm, pH 3–10). The IPG strips were equilibrated in buffer and subjected to SDS-PAGE using Criterion TGX gels (10–20%). For the second dimension, proteins were separated on 15% SDS polyacrylamide gels and followed by staining with Imperial Protein Stain overnight. Gels were destained using deionized water, imaged with a GS-800 Calibrated Densitometer (Bio-Rad Laboratories, USA), and analyzed using ImageMaster 2D Platinum 7.0 software (GE Healthcare). The spots showing intensity more than two-fold and reproducible changes in replicates were excised and subjected to in-gel digestion using trypsin^57^. Peptides were subjected to matrix-assisted laser desorption/ionization-time of flight (MALDI-TOF) mass spectrometric (MS) analysis (Microflex™ LT series, Bruker Daltonics, Germany). Peptide mass fingerprint (PMF) spectra were searched combined with MASCOT program search engine (http://www.matrixscience.com) and National Center for Biotechnology Information (NCBI) protein database (http://www.ncbi.nlm.nih.gov). Protein identities were confirmed by matching molecular weight (MW) and isoelectric point (pI) obtained from the 2-D gel electrophoresis.

### Metal measurements

For metal quantification, Inductively Coupled Plasma Emission Spectroscopy (ICP-ES) method has been used. Root and shoot of **s**eedlings were separated, washed thoroughly with sterile deionized nano-pure water, air dried for week, and then dried at 65 ^o^C in hot air oven for three days. About 25 to 30 mg of samples were digested in 5 mL conc. HNO_3_ at 80 ^o^C in Pyrex tubes for three days. The elements such as P, K, S, Mg, B, Co, Cu, Fe, Zn, and Mn were analyzed using ICAP *6000* Series ICP Emission Spectrometer (Thermo Scientific).

### Statistical analysis

Experiments were carried out under completely randomized design and repeated at least thrice. Data was collected randomly from many replicates or at least three replicates where indicated. The data were compared by the Student’s unpaired t-test *P* ≤ 0.05–0.001, and the presented as ±SEM.

## Additional Information

**How to cite this article**: Tiwari, M. *et al.* Comparative transcriptome and proteome analysis to reveal the biosynthesis of gold nanoparticles in Arabidopsis. *Sci. Rep.* **6**, 21733; doi: 10.1038/srep21733 (2016).

## Supplementary Material

Supplementary Information

## Figures and Tables

**Table 1:** Comparison of microarray data by quantitative RT-PCR.

Locus ID	Annotation	Microarray	qRT-PCR	25 ppm Au (Fold change)
		(Fold change)	10 ppm Au (Fold change)	
AT5G23990	FRO5	17.54	7.269	1.855
AT2G46830	CCA1	3.88	4.645	0.472
AT1G63950	HMA3	3.833	26.459	0.025
AT3G28345	ABC transporter B family member	3.102	2.215	0.526
AT5G14570	High affinity nitrate transporter	3.4	2.250	2.406
AT3G46900	COPT2	4.22	4.835	1.192
AT1G01060	LHY	3.59	2.978	1.638
AT3G12320	Light-inducible and clock-regulated 3, LNK3	4.81	8.889	0.498
AT1G33730	CYP76C5	3.77	1.685	2.308
AT5G17300	RVE1	2.919	2.689	1.986
AT5G24380	YSL2	3	1.332	0.386
AT3G09600	Myb	2.37	2.087	2.288
AT4G10530	Subtilase family protein	−6.85	0.073	0.450
AT5G49770	Leucine-rich repeat receptor-like protein kinase	−2.79	0.183	0.385
AT2G44840	ERF13	−2.01	0.179	1.116

**Table 2:** Au responsive proteins in root and shoot of *Arabidopsis*.

Spot ID	Tissue	Fold change	Protein	Score	Nominal mass (Da)	Calculated pI	Sequence coverage
85	Root	7.98	Putative inactive cadmium/zinc-transporting ATPase HMA3	34	59440	7.44	10%
33	Root	4.76	Thioredoxin-like protein HCF164, chloroplastic	23	28916	5.26	6%
4X	Root	3.50	UDP-glycosyltransferase 90A1	27	54055	5.65	5%
56	Root	2.99	Probable fructokinase-1	52	35424	5.31	20%
67	Root	2.56	Glyceraldehyde-3-phosphate dehydrogenase GAPC1, cytosolic	55	37028	6.62	27%
7X	Root	2.55	UDP-glycosyltransferase 73C5	31	56390	5.38	8%
36	Root	2.54	High mobility group B protein 7	33	27136	4.83	10%
21	Root	−2.19	Superoxide dismutase [Mn] 1, mitochondrial	32	25485	8.47	41%
28	Root	−2.73	Glutathione S-transferase F6	12	23471	5.8	15%
12	Root	−3.62	40S ribosomal protein S15-6	33	18849	10.59	21%
71	Root	−5.67	Vesicle-associated protein 2−2	26	43527	6.54	27%
67	Shoot	6.07	Thioredoxin domain-containing protein PLP3B	28	26522	5.54	9%
43	Shoot	5.50	Pentatricopeptide repeat-containing protein At1g62350	27	23386	5.05	23%
101	Shoot	4.02	Glycerophosphodiester phosphodiesterase GDPD3	31	42665	5.12	21%
63	Shoot	3.47	Glutathione S-transferase U21	20	25857	5.42	18%
85	Shoot	3.24	Probable 3-hydroxyisobutyrate dehydrogenase-like 3, mitochondrial	42	33549	6.34	27%
65	Shoot	3.00	Glutathione S-transferase U15	30	26689	5.46	21%
13	Shoot	2.90	U11/U12 small nuclear ribonucleoprotein 25 kDa protein	34	18775	9.75	32%
42	Shoot	2.89	Lactoylglutathione lyase	20	20892	5.14	15%
46	Shoot	2.72	Glutathione S-transferase F2	65	24114	5.92	37%
37	Shoot	2.60	PRA1 family protein G2	43	20633	5.15	24%
4	Shoot	2.55	NADH dehydrogenase [ubiquinone] 1 beta subcomplex subunit 9	38	13665	8.74	58%
7	Shoot	2.44	Cytochrome c oxidase subunit 5b-1, mitochondrial	47	19575	4.91	20%
98	Shoot	2.43	F-box protein PP2-A15	22	34631	6.16	21%
34	Shoot	2.00	Auxin-responsive protein IAA5	20	18741	6.37	41%
107	Shoot	−3.44	Serine racemase	27	35524	6.95	23%
