# Supplementary material for: Comparative transcriptome and proteome analysis to reveal the biosynthesis of gold nanoparticles in Arabidopsis
Source: Sci Rep. 2016 Feb 23;6:21733. doi: 10.1038/srep21733 (PMC4763195; doi:10.1038/srep21733)
Supplement: Supplementary Information [file srep21733-s1.pdf]

Comparative transcriptome and proteome analysis to reveal the biosynthesis of gold nanoparticles in Arabidopsis

Manish Tiwari, Sneha Krishnamurthy, Devesh Shukla, Jeffrey Kiiskila[1], Ajay Jain[2], Rupali Datta[1], Nilesh Sharma, Shivendra V. Sahi*

Department of Biology, Western Kentucky University, 1906 College heights, Bowling Green, 42101-1080 Kentucky, USA

[1]Department of Biological Sciences, Michigan Technological University, Houghton, Michigan, USA

[2]National Research Centre on Plant Biotechnology, Lal Bahadur Shastri Building, Pusa Campus, New Delhi, 110012, India

*****Corresponding Author; **Phone**: 270 745-6012 **Fax**: 270 745-6856

E-mail: shiv.sahi@wku.edu

Supplementary Figure 1: GO term enrichment of differentially expressed genes

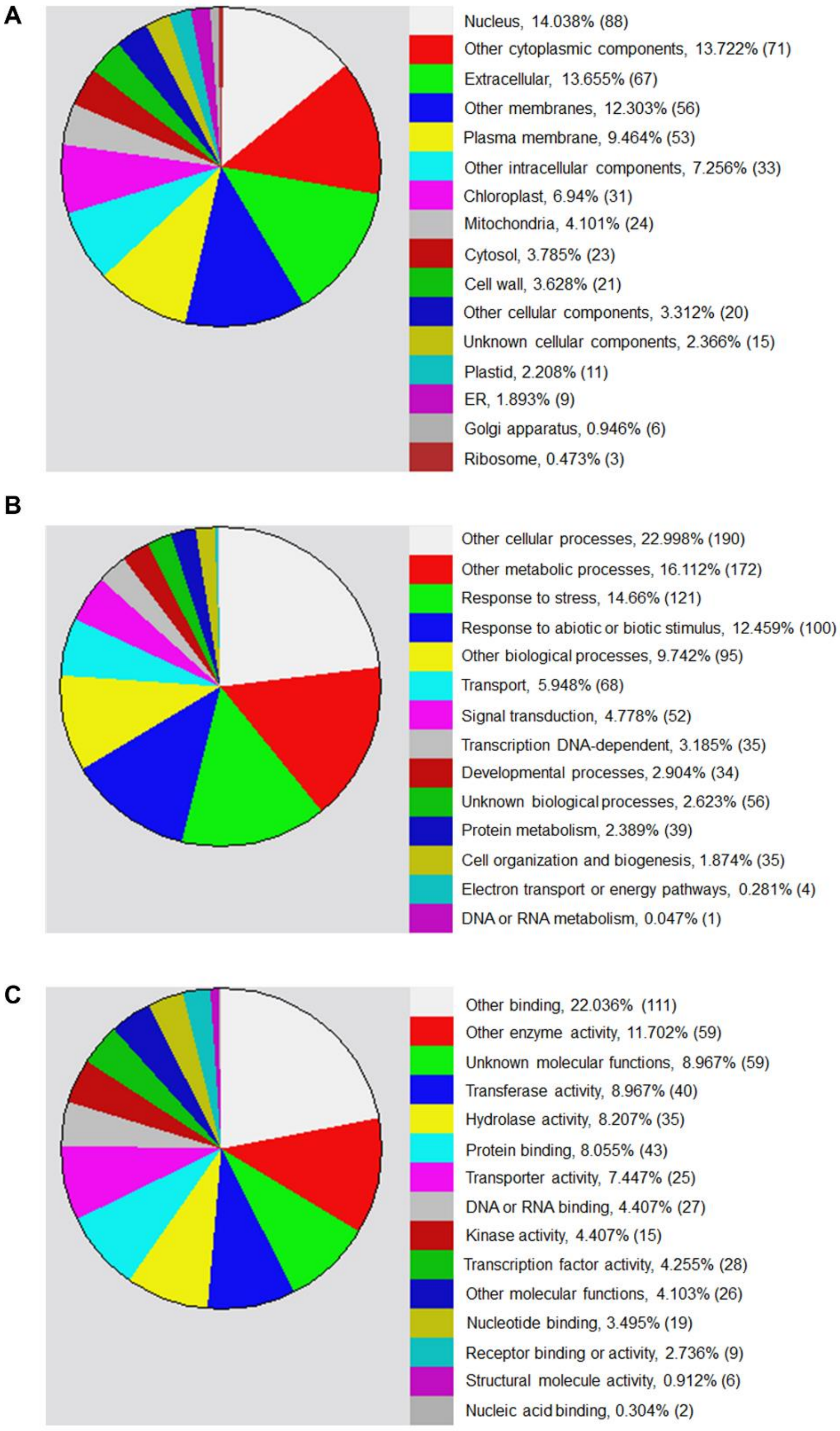

Supplementary Figure 2: Distribution of upregulated genes based on metal responsive nature

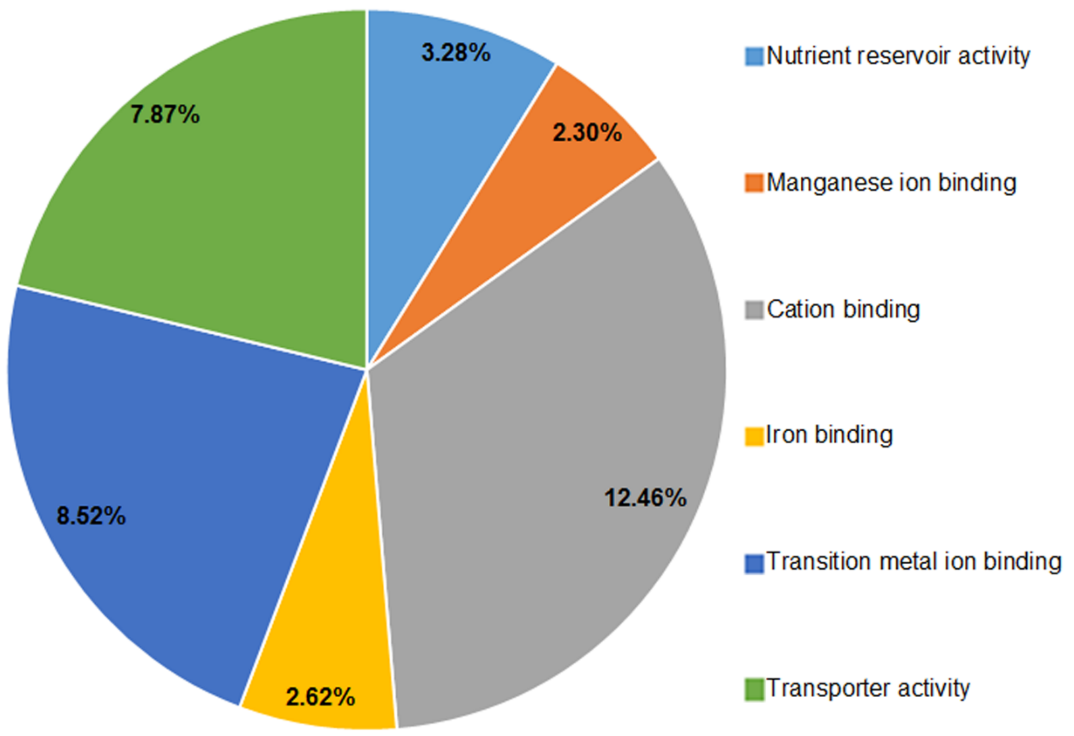

Supplementary Table 1: GO slim molecular function categorization of upregulated genes through Plant Gene Set Enrichment Analysis

Gene set name (no. of genes)	Description	Gene number in overlap	P value	FDR
Iron ion binding (499)	GO:0005506 iron ion binding, GO slim:molecular function	8	3.13e-05	0.0214
Transcription regulator activity (1738)	GO:0030528 transcription regulator activity, GO slim:molecular function	14	6.85e-05	0.0234
Oxidoreductase activity, oxidizing metal ions, NAD or NADP as acceptor (8)	GO:0016723 oxidoreductase activity, oxidizing metal ions, NAD or NADP as acceptor, GO slim:molecular function	2	2.58e-4	0.0293
Ferric chelate reductase activity (8)	GO:0000293 ferric-chelate reductase activity, GO slim:molecular function	2	2.58e-4	0.0293
Oxidoreductase activity, acting on paired donors, with incorporation or reduction of molecular oxygen (461)	GO:0016705 oxidoreductase activity, acting on paired donors, with incorporation or reduction of molecular oxygen, GO slim:molecular function	7	1.41e-4	0.0293

Supplementary Table 2: GO slim molecular function categorization of downregulated genes through Plant Gene Set Enrichment Analysis

Gene set name (no. of genes)	Description	Gene number in Overlap	P value	FDR
Nutrient reservoir activity (67)	GO:0045735 nutrient reservoir activity, GO slim:molecular_function	9	2.63e-11	3.11e-08
Manganese ion binding (46)	GO:0030145 manganese ion binding, GOslim:molecular_function	7	2.09e-09	1.23e-06
Ion binding (3006)	GO:0043167 ion binding, GOslim:molecular_function	33	1.04e-07	3.09e-05
Cation binding (3005)	GO:0043169 cation binding, GO slim:molecular_function	33	1.04e-07	3.09e-05
O-methyltransferase activity (34)	GO:0008171 O-methyltransferase activity, GOslim:molecular_function	5	5.38e-07	1.27e-4
Metal ion binding (2829)	GO:0046872 metal ion binding, GOslim:molecular_function	30	9.78e-07	1.93e-4
Chitinase activity (24)	GO:0004568 chitinase activity, GO slim:molecular_function	4	5.02e-06	8.48e-4
Heme binding (373)	GO:0020037 heme binding, GOslim:molecular_function	9	2.83e-05	4.18e-3
Catalytic activity (8725)	GO:0003824 catalytic activity, GO slim:molecular_function	57	3.31e-05	4.35e-3
Tetrapyrrole binding (406)	GO:0046906 tetrapyrrole binding, GO slim:molecular_function	9	5.37e-05	6.35e-3
Transition metal ion binding (2298)	GO:0046914 transition metal ion binding, GO slim:molecular_function	22	1.63e-4	0.0175
Iron ion binding (499)	GO:0005506 iron ion binding, GO slim:molecular_function	9	2.46e-4	0.0242

Antioxidant activity (142)	GO:0016209 antioxidant activity, GO slim:molecular_function	5	3.51e-4	0.0297
Electron carrier activity (522)	GO:0009055 electron carrier activity, GOslim:molecular_function	9	3.4e-4	0.0297
Binding (11174)	GO:0005488 binding, GO slim:molecular_function	64	5.08e-4	0.0401

Supplementary Table 3: List of Au responsive upregulated genes in microarray analysis

S.N.	Locus ID	Gene Annotation	Fold-Change
1.	AT5G23990	Ferric reduction oxidase 5	17.5377
2.	AT1G52820	2-oxoglutarate-dependent dioxygenase	6.10238
3.	AT5G04120	Phosphoglycerate mutase-like protein	5.37988
4.	AT1G18870	Isochorismate synthase 2	4.97757
5.	AT1G71200	Basic helix-loop-helix domain-containing protein	4.97558
6.	AT3G12320	Light-inducible and clock-regulated 3, LNK3	4.80791
7.	AT1G26790	Dof-type zinc finger domain-containing protein	4.33402
8.	AT3G46900	Copper transporter 2	4.21698
9.	AT5G14570	High affinity nitrate transporter 2.7	3.8982
10.	AT2G46830	CCA1, Myb family	3.87975
11.	AT1G63950	Heavy-metal-associated domain-containing protein	3.83307
12.	AT4G28790	bHLH23 transcription factor	3.78458
13.	AT1G33730	Cytochrome P450, family 76, C5, CYP76C5	3.77075
14.	AT2G16005	MD-2-related lipid recognition domain-containing protein	3.70453
15.	AT1G01060	Late elongated hypocotyl protein, Myb family	3.59428
16.	AT5G23980	Ferric reduction oxidase 4	3.56527
17.	AT5G59520	Zinc transporter 2	3.31975
18.	AT4G21680	Nitrate transporter 1.8	3.26447
19.	AT2G38255	Hypothetical protein	3.24975
20.	AT3G28345	ABC transporter B family member 15	3.102
21.	AT5G24380	Metal-nicotianamine transporter YSL2	2.9997
22.	AT5G03545	Hypothetical protein	2.99139
23.	AT5G17300	Reveille 1 protein, Myb family	2.91968
24.	AT1G47510	Inositol polyphosphate 5-phosphatase 11	2.87279
25.	AT5G15948	S-adenosylmethionine decarboxylase-like protein	2.79225
26.	AT3G22550	Hypothetical protein	2.76846
27.	AT2G31380	Salt tolerance-like protein	2.72006
28.	AT2G46790	Two-component response regulator-like APRR9	2.70904
29.	AT4G22210	Defensin-like protein 97	2.7019
30.	AT1G06100	Delta-9 desaturase-like 2 protein	2.67852
31.	AT1G07610	Metallothionein 1C	2.63142
32.	AT4G11210	Dirigent domain-containing protein	2.60236
33.	AT5G54470	B-box type zinc finger-containing protein	2.5976
34.	AT5G06980	Hypothetical protein	2.59096
35.	AT1G75830	Defensin-like protein 13	2.55809
36.	AT5G37260	Reveille 2 protein, Myb family	2.53845
37.	AT1G18290	Hypothetical protein	2.51045
38.	AT1G34510	Peroxidase 8	2.4756
39.	AT4G25100	Superoxide dismutase [Fe]	2.44342
40.	AT1G08430	Aluminum-activated malate transporter 1	2.37952

41.	AT1G53540	HSP20-like chaperone	2.37592
42.	AT4G11880	Agamous-like MADS-box protein AGL14	2.36954
43.	AT3G09600	Myb family transcription factor	2.36651
44.	AT5G06900	Cytochrome P450, family 93, subfamily D	2.31686
45.	AT5G36130	Cytochrome P450 family protein	2.30527
46.	AT4G11190	Dirigent domain-containing protein	2.26673
47.	AT3G09922	Induced by phosphate starvation1	2.2666
48.	AT2G31083	CLAVATA3 protein	2.25834
49.	AT1G68740	Phosphate transporter PHO1-1	2.25428
50.	AT3G13840	Scarecrow-like protein 29	2.24667
51.	AT5G62430	Dof zinc finger protein DOF5.5	2.24577
52.	AT5G25460	Hypothetical protein	2.23767
53.	AT4G13390	Proline-rich extensin-like family protein	2.19473
54.	AT3G49620	2-oxoglutarate-Fe(II)-dependent oxygenase	2.16134
55.	AT2G25160	Cytochrome P450, family 82, subfamily F	2.15761
56.	AT3G16800	Protein phosphatase 2C 41	2.14452
57.	AT1G60450	Galactinol synthase 7	2.1428
58.	AT2G21210	SAUR-like auxin-responsive protein	2.11956
59.	AT5G63190	MA3 domain-containing protein	2.09843
60.	AT5G48010	Thalianol synthase 1	2.08992
61.	AT3G47420	Phosphate starvation-induced protein	2.08306
62.	AT5G02020	Hypothetical protein	2.07093
63.	AT2G24980	Proline-rich extensin-like family protein	2.06656
64.	AT1G23450	Pentatricopeptide repeat-containing protein-like protein	2.03601
65.	AT4G13420	Potassium transporter 5	2.0341
66.	AT2G25150	HXXXD-type acyl-transferase-like protein	2.02808
67.	AT3G29780	Protein ralf-like 27	2.02344
68.	AT4G12490	Seed storage 2S albumin superfamily protein	2.01751
69.	AT4G15550	Indole-3-acetate beta-D-glucosyltransferase	2.00635
70.	AT4G02170	Hypothetical protein	2.00502

Supplementary Table 4: List of Au responsive downregulated genes in microarray analysis

S.N.	Locus ID	Gene Annotation	Fold-Change
1.	AT4G31970	Cytochrome P450, family 82, subfamily C	-13.2573
2.	AT5G38910	Germin-like protein subfamily 1 member 9	-12.8769
3.	AT5G40990	GDSL esterase lipase 1	-12.5087
4.	AT2G30750	Cytochrome P450 71A12	-11.2062
5.	AT3G05950	Germin-like protein subfamily 1 member 7	-10.8316
6.	AT1G26240	Proline-rich extensin-like family protein	-10.3365
7.	AT3G60270	Uclacyanin	-9.85822
8.	AT2G30660	3-hydroxyisobutyryl-CoA hydrolase	-8.3746
9.	AT4G13300	Terpenoid synthase 13	-7.67573
10.	AT5G42530	Hypothetical protein	-7.59356
11.	AT2G43000	NAC domain-containing protein 42	-7.20664
12.	AT4G10530	Subtilase family protein	-6.85105
13.	AT2G30770	Cytochrome P450, family 71, subfamily A	-6.63149
14.	AT1G15125	S-adenosyl-L-methionine-dependent methyltransferase	-6.52484
15.	AT2G02930	Glutathione S-transferase 16	-6.44904
16.	AT3G03670	Peroxidase	-6.28715
17.	AT5G52710	Copper transport family protein	-5.85748
18.	AT1G21120	O-methyltransferase family protein	-5.77294
19.	AT5G52670	Copper transport protein	-5.73767
20.	AT5G44585	Hypothetical protein	-5.425
21.	AT5G57220	Cytochrome P450, family 81, subfamily F	-5.26538
22.	AT5G64120	Peroxidase 71	-5.22024
23.	AT1G26410	FAD-binding and BBE domain-containing protein	-5.207
24.	AT4G19760	Glycosyl hydrolase family protein	-5.15035
25.	AT5G39120	Germin-like protein subfamily 1 member 15	-5.07603
26.	AT1G21110	O-methyltransferase family protein	-4.87295
27.	AT4G14630	Germin-like protein subfamily 1 member 8	-4.76824
28.	AT1G74080	Myb domain protein 122	-4.59534
29.	AT1G57630	Toll-Interleukin-Resistance domain-containing protein	-4.50851
30.	AT4G19750	Glycosyl hydrolase family protein	-4.49295
31.	AT2G43510	Defensin-like protein 195	-4.35663
32.	AT1G31580	ECS1 protein	-4.30711
33.	AT4G18250	Receptor serine threonine kinase	-4.26378
34.	AT5G60100	Two-component response regulator-like APRR3	-4.21684
35.	AT3G12230	Serine carboxypeptidase-like 14	-4.13825
36.	AT1G08090	Nitrate transporter 2:1	-4.10797
37.	AT1G21130	O-methyltransferase-like protein	-4.07761
38.	AT1G02920	Glutathione S-transferase 11	-4.03949
39.	AT2G43570	Chitinase class 4-like protein	-3.9401
40.	AT1G05880	E3 ubiquitin-protein ligase ARI12	-3.92679

41.	AT4G15990	Hypothetical protein	-3.90724
42.	AT4G16860	TIR-NBS-LRR class protein	-3.89922
43.	AT5G48430	Aspartyl protease family protein	-3.87418
44.	AT4G04990	Hypothetical protein	-3.85765
45.	AT4G19720	Glycosyl hydrolase family protein	-3.746
46.	AT1G67980	Caffeoyl-CoA 3-O-methyltransferase	-3.71791
47.	AT2G33770	Ubiquitin-conjugating enzyme E2	-3.68801
48.	AT1G21230	Wall-associated receptor kinase 5	-3.65012
49.	AT1G08830	Superoxide dismutase (Cu-Zn)	-3.62763
50.	AT4G36410	Ubiquitin-conjugating enzyme E2 17	-3.58101
51.	AT2G38600	HAD superfamily, subfamily IIIB acid phosphatase	-3.52082
52.	AT2G26560	Phospholipase A 2A	-3.51641
53.	AT2G30670	Tropine dehydrogenase	-3.4913
54.	AT1G18970	Germin-like protein subfamily T member 1	-3.46163
55.	AT5G38540	Jacalin lectin family protein	-3.43039
56.	AT1G26250	Proline-rich extensin-like family protein	-3.38242
57.	AT5G47240	Nudix hydrolase 8	-3.38169
58.	AT5G59680	Leucine-rich repeat protein kinase-like protein	-3.37181
59.	AT4G23700	Cation H(+) antiporter 17	-3.36662
60.	AT5G26690	Heavy-metal-associated domain-containing protein	-3.22911
61.	AT2G21900	WRKY transcription factor 59	-3.22178
62.	AT4G10860	Hypothetical protein	-3.15932
63.	AT1G02930	Glutathione S-transferase 1	-3.12612
64.	AT5G43650	bHLH92	-3.10083
65.	AT5G42830	HXXXD-type acyl-transferase-like protein	-3.08226
66.	AT5G41080	Glycerophosphoryl diester phosphodiesterase protein	-3.07135
67.	AT2G23270	Hypothetical protein	-2.9786
68.	AT1G64370	Hypothetical protein	-2.94879
69.	AT5G01900	WRKY transcription factor 62	-2.92625
70.	AT1G52130	Mannose-binding lectin-like protein	-2.9198
71.	AT4G14680	3'-phosphoadenosine 5'-phosphosulfate synthase	-2.8995
72.	AT2G40080	Hypothetical protein	-2.89458
73.	AT1G64170	Cation H(+) antiporter 16	-2.89
74.	AT5G40010	AAA-ATPase 1	-2.86254
75.	AT1G26380	FAD-binding and BBE domain-containing protein	-2.85807
76.	AT5G25250	Flotillin-like protein 1	-2.85524
77.	AT5G11920	beta-fructofuranosidase	-2.85436
78.	AT2G23030	Serine threonine-protein kinase, SRK2J	-2.85078
79.	AT1G14540	Peroxidase 4	-2.81827
80.	AT5G56960	Putative transcription factor bHLH041	-2.81712
81.	AT5G52720	Copper transport family protein	-2.81608
82.	AT2G39400	Alpha beta-hydrolase domain-containing protein	-2.80153
83.	AT1G32350	Alternative oxidase 3	-2.79736

84.	AT5G49770	Leucine-rich repeat receptor-like protein kinase	-2.7882
85.	AT5G39150	Germin-like protein subfamily 1 member 17	-2.78272
86.	AT5G14760	L-aspartate oxidase	-2.78187
87.	AT4G33050	Calmodulin-binding protein	-2.74687
88.	AT1G79680	Wall-associated receptor kinase-like 10	-2.74338
89.	AT4G24350	Phosphorylase family protein	-2.7329
90.	AT4G27850	Glycine-rich family protein	-2.73115
91.	AT1G04370	Ethylene-responsive transcription factor 14	-2.70828
92.	AT5G19890	Peroxidase 59	-2.69694
93.	AT3G56950	Galactinol--sucrose galactosyltransferase 2	-2.69226
94.	AT1G67870	Glycine-rich protein	-2.67387
95.	AT5G21960	Ethylene-responsive transcription factor ERF016	-2.64697
96.	AT1G30370	Lipase-3 domain-containing protein	-2.63567
97.	AT1G19250	Flavin-containing monooxygenase 1	-2.61705
98.	AT3G28510	AAA-type ATPase family protein	-2.60891
99.	AT5G44990	Glutathione S-transferase family protein	-2.60543
100.	AT1G19610	Defensin-like protein 19	-2.60311
101.	AT5G46050	Peptide transporter PTR3-A	-2.60253
102.	AT5G38940	Germin-like protein subfamily 1 member 11	-2.59104
103.	AT3G01175	Hypothetical protein	-2.56899
104.	AT4G22690	Cytochrome P450, family 706, subfamily A	-2.56516
105.	AT2G33830	Dormancy auxin associated protein	-2.56427
106.	AT1G21100	O-methyltransferase-like protein	-2.5576
107.	AT4G24015	RING-H2 zinc finger protein RHA4a	-2.54901
108.	AT1G33790	Jacalin lectin family protein	-2.54856
109.	AT3G63380	Ca2+-transporting ATPase	-2.54321
110.	AT1G33900	Avirulence-responsive protein	-2.53974
111.	AT1G51920	Hypothetical protein	-2.5385
112.	AT5G02780	Glutathione transferase lambda 1	-2.5356
113.	AT4G37290	Hypothetical protein	-2.53243
114.	AT4G32950	Protein phosphatase 2C 61	-2.53042
115.	AT5G02490	Heat shock protein 70	-2.5276
116.	AT1G54950	Hypothetical protein	-2.51776
117.	AT3G12580	Heat shock protein 70-4	-2.50915
118.	AT5G13320	Auxin-responsive GH3 family protein	-2.50371

Supplementary Table 5: *cis*- regulatory motif prediction in 1 kb upstream sequences of GSTs through Plant Cis-acting Regulatory DNA Elements

Promoter	Locus	Site name	Position	Strand	Sequence	Function
GSTF6	AT1G02930	ABRE	87	+	TACGGTC	cis-acting element involved in the abscisic acid responsiveness
		W box	698	+	TTGACC	
GST11	AT1G02920	W box	285	+	TTGACC	
		W box	728	-	TTGACC	
		MBS	87	+	TAACTG	MYB binding site involved in drought-inducibility
GST16	AT2G02930	ABRE	60	+	ACGTGGC	cis-acting element involved in the abscisic acid responsiveness
		MRE	497	-	AACCTAA	MYB binding site involved in light responsiveness
GSTL1	AT5G02780	MBS	750	+	CAACTG	MYB binding site involved in drought-inducibility
		W box	27<br>826	+	TTGACC	
GST	AT5G44990	W box	806	-	TTGACC	

Supplementary Table 6: List of details about oligonucleotides with sequences used in the study

S.N.	Primer name	Primer sequences (5’-3’)
1.	FRO5 F	GACCCCCAAACTCGCCAAGCAT
2.	FRO5 R	TCATCACCCTCCCCATTCTCCC
3.	CCA1 F	ACGTGAAAGGTGGACTGAGGAA
4.	CCA1 R	GCTTCAGCCTCTTTCTCTACCTTGG
5.	NRT2.7 F	TGGCTGGGTTGTTGTGCGTG
6.	NRT2.7 R	CCTAACGATCGCGTGGAGACG
7.	LHY F	TCGGCCTCTTCTTCACAGTTGA
8.	LHY R	CGCCACTTACCTGTTTCGTTGGT
9.	CYP76C5 F	CCTCCCAAGAAAAGCGGAAAGGG
10.	CYP76C5 R	TGGGTCTCGTCCTATGGCCC
11.	YSL2 F	TATCCTAGTGGAACGGCAACAGC
12.	YSL2 R	AAACCCGCGAATCTGTTTCTTGGC
13.	RVE1 F	GCGTCGTCTCCGTTGACTGC
14.	RVE1 R	GGCTTCCGTACCTTGGGTGC
15.	ERF13 F	GCTGGACACCCTCCGTTCCT
16.	ERF13 R	CACGGCCTCCTCCTCACTCC
17.	COPT2 F	CCTCCTCGCCGTAATTGCCGA
18.	COPT2 R	CACAGCGGTTTGAGCGAGCC
19.	MYB F	GGCATGGGAAGCTCAAGCCG
20.	MYB R	AGGAACACCGTGAAGCACTGGA
21.	LRR-RLK F	TTTGCCAAGTGGGATGCGGG
22.	LRR-RLK R	GGCCATAACCTCCACCGCCA
23.	LNK3 F	GTCTTTCAAGGAGGAAGCTCCA
24.	LNK3 R	CCCGGAAACATTTGCGTGTCT
25.	ABCB F	TGGTGCTCAGGGTGGTACTGT
26.	ABCB R	ACCACCACCAAGTGATACGCCA
27.	SUBTIL F	ATGGCACGCATGTTGCCTCG
28.	SUBTIL R	ACATCAGCTCCCGAACACCCT
29.	HMA3 F	CTGACTTACCCGGAATTCACTC
30.	HMA3 R	TCCCTCTTCTGCTTAGCCTCA
